# Duchenne Muscular Dystrophy Presenting as Incidental Hyper-Transaminasasemia in a Two-Month-Old Male

**DOI:** 10.7759/cureus.35498

**Published:** 2023-02-26

**Authors:** Uzoego N Chibuzo, Madeline Bruman, Ariela Holguin, Babu Bangaru

**Affiliations:** 1 Department of Pediatrics, Flushing Hospital Medical Center, New York, USA; 2 Department of Pediatrics, Columbia University Irving Medical Center, Vagelos College of Physicians and Surgeons, New York, USA; 3 Department of Pediatrics, American University of the Caribbean School of Medicine, Cupecoy, SXM; 4 Department of Pediatrics, New York University, School of Medicine, New York, USA; 5 Pediatric Gastroenterology, Children's Hospital of Los Angeles, Los Angeles, USA

**Keywords:** cardiorespiratory failure, proximal muscle weakness, cardioprotective interventions, newborn screen, cardiomyopathy, dystrophin, x linked disease, creatine kinase, hyper-transaminasasemia, duchenne’s muscular dystrophy

## Abstract

Duchenne's muscular dystrophy (DMD) is a debilitating X-linked recessive disorder of dystrophin gene expression that culminates in the downregulation of dystrophin in cardiac and skeletal muscle. As a result, there is progressive muscle weakness, fibrosis, and atrophy. The skeletal and cardiac muscle degeneration rapidly progresses to the respective loss of ambulation and death from cardiac muscle failure by the second and fourth decades of life. Although muscle degeneration has been demonstrated in utero patients are initially asymptomatic. Therefore, diagnosis is typically delayed until about five years of age when proximal muscle weakness initiates a diagnostic workup that uncovers the disease. We present the rare case of an early diagnosis of DMD.

A two-month-old, the only male offspring of a family with three children, was discovered to have hyper-transaminisemia during hospitalization for pneumonia. His preceding medical history was only significant for fever, cough, and rhinorrhea. The pregnancy and birth were uneventful. No abnormalities were detected on the newborn screen. Physical examination was reassuring with no peripheral stigmata of liver disease. Ultrasonographic assessments, metabolic assays, and infectious disease markers were within normal limits. Creatine kinase (CK) was markedly elevated and our patient was subsequently confirmed to be positive for a pathogenic hemizygous variant of the DMD gene.

Reliance on an abnormal clinical presentation to trigger diagnostic workup for DMD has led to delays in the diagnosis of this genetic disorder. Incorporating CK analysis into newborn screening panels may enable more children to commence workup in infancy rather than at the current average age of 4.9 years. Early diagnosis is of value in the early initiation of monitoring, anticipatory guidance, and availing families’ opportunities to harness current trends of care.

## Introduction

Duchenne’s muscular dystrophy (DMD) is an X-linked recessive disorder affecting the dystrophin gene [1]. It is the most common muscular dystrophy seen in children worldwide [1]. Despite the presence of dystrophin phenotypes in the early embryo, the mean age at which the disease is diagnosed is 4.9 years [2]. We present a male infant who was diagnosed early with the disease after he was incidentally found to have persistent hyper-transaminasemia.

## Case presentation

Our two-month-old male patient presented to the pediatric gastroenterology clinic with persistent hyper-transaminasemia (Table 1). The elevated serum transaminase levels were first discovered during hospitalization for pneumonia when he was two months old. His past medical history was significant for neonatal jaundice at 30 hours of life which was resolved with phototherapy. He had two episodes of cough, fever, and rhinorrhea managed with saline sprays and nebulization at home. He was the product of an uncomplicated pregnancy and birth. No abnormalities were detected in the newborn screen. He was fully vaccinated and his parameters for growth and development were normal. He was the only male offspring of his parents’ three children. The family history was only notable for asthma in the maternal grandmother, there was no consanguinity.

**Table 1:** Serum Transaminase Assay PNA= pneumonia, HA= hospital admission, PCP= primary care physician, PD = Post discharge, FU= Follow-up, GI= Gastroenterology, SC = Specialist Clinic, No= Number.

Encounter	Alanine Aminotransferase (ALT)	Aspartate Aminotransferase (AST)
	(ALT Normal Lab Interval: 21- 71 IU/L)	(AST Normal Lab Interval: 17- 59 IU/L)
PNA HA	248 IU/L	382 IU/L
PCP PD FU	304 IU/L	424 IU/L
GI SC Visit No 1	313 IU/L	422 IU/L
GI SC Visit No 2	249 IU/L	327 IU/L

Laboratory investigations ordered on the first visit excluded Toxoplasma, Cytomegalovirus, Hepatitis A, B, and E Infections, and the coagulation profile was found to be normal. The level of aspartate aminotransferase (AST) and alanine aminotransferase (ALT) were elevated. The right upper abdominal quadrant ultrasound scans were unremarkable. He was referred to a pediatric ophthalmologist for a comprehensive eye examination which detected no abnormality.

During the second pediatric gastroenterology clinic visit, at the age of four months, only cough and rhinorrhea were noted. Investigations ordered during the visit revealed normal growth hormone, galactose -1- phosphate, alpha 1 antitrypsin, amino acid, and urine organic acid assays. Serum transaminases remained elevated (Table 1). The complete abdominal ultrasound scan was normal. Creatine kinase (CK) levels were found to be markedly elevated and prompted a muscular dystrophy workup.

He was referred to pediatric neurology, physical medicine, and rehabilitation. With the onset of subtle physician-identified motor delay, he was subsequently enrolled in the early intervention program. A social work referral was also made. The Spinal Muscular Atrophy Panel was negative. Multi-gene panel testing for Neuromuscular Disorders revealed a hemizygous pathogenic variant in DMD gene exon 6, c.9100C>T (p. Arg3034).

## Discussion

DMD is an X-linked recessive disease that occurs in 15.9 cases per 100,000 male live births in the USA and one in 3,600 male live-born infants worldwide [1-3]. The disease results from the mutation in the Dystrophin gene (locus Xp21.2) and consequent Dystrophin deficiency. The dystrophin glycoprotein complex of the cytoskeleton creates a mechanical link between the actin of the myofibrils and the extracellular matrix, which is vital in the protection of muscles during contraction. Dystrophin-deficient myofibrils rupture during contraction with subsequent calcium infiltration and activation of calcium-dependent Caspian proteases which cause muscle necrosis. These muscles then undergo recurring cycles of necrosis, regeneration failure, and fibrosis which culminate in the clinical presentation of progressive muscle degeneration, loss of ambulation, assisted ventilation, cardiorespiratory failure, and premature death [3-4].

Typically, initial clinical presentation for nonfamilial DMD occurs at 3.6 years following a year of caregivers’ observation of concerning symptoms such as toe walking, clumsiness, or a positive Gower’s sign. In the latter sign, to stand up from a sitting position, the child flexes the trunk at the hips, places their hands on their knees, then progressively extends the trunk by using the hands to "climb" up their legs. These concerning symptoms initially prompt referral to physical or occupational therapists and developmental stimulation programs. Usually, a year later, neuromuscular specialists’ referrals lead to CK analysis and the subsequent diagnosis of the disease. CK values, essential in relating the patient’s presentation to an underlying muscular disorder, are first ordered at a mean age of 4.9 years [2,5]. In contrast, our patient’s CK analysis was performed four months after hyper-transaminasemia prompted a gastroenterology referral. The timely initiation of investigations to eliminate hepatic and non-hepatic etiologies led to one of the earliest reported ages for the diagnosis of non-familial DMD [2].

Concerns have been expressed about diagnostic delays in DMD which have culminated in a mean age of diagnosis of 4.9 years [2]. Newborn screening presents a logical means of detecting DMD that has demonstrated phenotypic expression in studied pluripotent human cells as early as the somite stage of the embryo. In the 1970s, a newborn screen of patients with DMD was explored in many countries [5]. However, in the absence of validated simple screening tests as well as early treatment interventions that could be instituted to improve the outcome of DMD diagnosed in the neonatal period, most newborn screening studies for DMD were discontinued [5-6]. In the USA, DMD screening is not incorporated into the Recommended Uniform Screening Panel (RUSP) [7]. However, the FDA approved kits that enable the incorporation of DMD into Newborn panels in 2019. Studies have indicated the benefit of pre-symptomatic therapy initiation [8-9]. Thus, the establishment of the diagnosis of DMD at the earliest possible juncture is of increasing relevance today. Even in the absence of definitive interventions, early diagnosis in patients has demonstrated psychosocial benefits in quality of life as well as relevance in DMD recurrence prevention, as an estimated 15 to 20 percent of all DMD cases are potentially prevented through reproductive planning and prenatal testing [6].

In patients with neuromuscular disease (NMD), deterioration of respiratory muscles results in decreased pre-cough inspiration, impaired cough, and airway clearance [10-11]. Respiratory tract infections further compromise the clinical presentation leading to impaired clearance of secretions, mucous plugging, atelectasis, respiratory insufficiency, and pneumonia [10-11]. Our patient had multiple respiratory presentations, including hospitalization for pneumonia during which his persistent hyper-transaminasemia was identified. However, an ascription of these presentations to the subtle manifestations of DMD, rather than a co-existing pathology, requires precise delineation of the natural history of DMD in infants. Unfortunately, the later age of diagnosis of DMD currently precludes the existence of a large cohort of infants that have been carefully monitored for early respiratory presentations of the disease [2,5]. In older children, respiratory disorders associated with DMD correlate to the progression of muscle weakness [12]. An anticipatory approach to respiratory management including monitoring of respiratory muscle function, timely employment of volume recruitment, assisted coughing, nocturnal and subsequently daytime ventilation is currently advocated in patients with DMD. Currently, it is predicted that with anticipatory respiratory management, children may start to be ventilated at an earlier age [13]. While studies in infants with other NMD such as Type I spinal muscular atrophy reveal that early initiation of assisted ventilation is associated with improved lung development and function, the ideal age for initiation of assisted ventilation in DMD requires more precise delineation [14]. Therefore, early diagnosis of the disease, as in our patient, becomes a necessity not only for establishing the natural history of the disease and monitoring function but also for identifying the earliest time at which assisted ventilation, lung volume recruitment and assisted coughing can optimally be employed.

Dystrophin deficiency manifests as cardiomyopathy in the heart [5]. Progressive fibrosis of the myocardium leads initially to asymptomatic cardiomyopathy and later to symptomatic ventricular failure, dilated cardiomyopathy, as well as life threatening ventricular arrhythmias [5]. Historically, due to the subtle signs and symptoms of their cardiomyopathy, particularly in non-ambulant patients, cardiac interventions were instituted late following a substantial decline in left ventricular ejection fraction [5]. Currently, more sensitive methods of monitoring, like cardiac magnetic resonance (CMR) with late gadolinium-enhancement imaging, enable the identification of myocardial damage prior to the onset of echocardiographic evidence of left ventricular (LV) dysfunction [5,15]. Interventional studies into cardio-protective therapies, such as the potassium-sparing diuretic eplerenon, revealed the greatest efficacy of the drug was demonstrated in younger children with consequent improvement in LV strain on CMR [8-9]. In contrast, older children only had an associated stabilization of their previously observed decline in myocardial function. Although the youngest child in the study was seven years old, each child already had evidence of myocardial damage. This, along with the demonstration of dystrophin phenotypes in the early embryo, suggests myocardial damage by CMR may be detected in even younger children than these. The early diagnosis in our asymptomatic patient has the potential for optimal monitoring and early identification of myocardial injury. Current research suggests that such asymptomatic patients may be better suited for cardioprotective interventions geared towards altering the natural history of decline in myocardial function [8-9,15].

DMD is also associated with a loss of intelligence quotient (IQ) [16]. More intellectual disability (Full Scale IQ <70) is associated with mutations in the distal portion of the DMD gene [16]. A significant increase in the IQ of children enrolled in early intervention programs has been demonstrated [17]. Currently, the mean age of diagnosis of DMD occurs after the age at which most children are eligible for enrollment in this program. With an exon 61 mutation, our patient also has an increased risk for intellectual disability, but his early diagnosis would permit him to utilize currently available early intervention programs to develop his intellectual potential more fully.

Prednisolone or deflazacort and physiotherapy are widely used interventions in DMD associated with improved cardiac and respiratory function as well as decreased incidence of scoliosis [18]. Various other drugs are the subject of trials including anti-inflammatory, antioxidant molecules, drugs that target myostatin, regulate utrophin, reduce fibrosis, improve vasodilatation and myocardial function [5,18]. The approval for the use of these drugs remains limited by patient selection for clinical trials [5]. Early diagnosis and early inclusion of patients such as ours in dystrophin registries can potentially harness patient selection for trials. Although there is currently no gene therapy for DMD, dystrophin restorative therapies with antisense oligonucleotides, amino acids, or peptide antibiotics are being explored [4]. The use of restorative therapies such as gentamicin for stop codon read through revealed increased dystrophin post gentamicin therapy in patients with higher pretreatment levels of dystrophin [19]. This in conjunction with animal studies that have demonstrated an age dependent decline in dystrophin levels may suggest the drug has greater utility when employed early in patients like ours without the overt disease [19]. Currently, eteplirsen and golodirsen have FDA approval for exon 51 and 53 skipping therapy [20]. Our patient’s mutation was at exon 61. However, the accelerated FDA approval of golodirsen underscores the value of early diagnosis in enabling the prompt identification of eligible patients for management with such specific targeted treatments.

## Conclusions

DMD is asymptomatic in its early presentation. Diagnosis largely occurs after primary caregivers note aberrations in ambulation or, less often, elevations in serum enzymes trigger further investigations for which elevated CK is pivotal in the diagnosis of the disease. Our current reliance on physical and biochemical aberrations to prompt investigations into DMD has led to diagnostic delays. Our patient deviates strikingly from the norm with CK levels detected very early at four months of age following extensive evaluation for hyper-transaminasemia. The inclusion of CK analysis into the RUSP can potentially eliminate multiple sources of diagnostic delays in children with DMD. Early diagnosis is of utmost importance to understand the natural history of the disease, facilitate the patient selection and enrollment vital to DMD trials, institute early monitoring, provide anticipatory guidance, and ensure no opportunities are missed to harness current trends of care.
